# Presentation of gastrointestinal bleeding in patients with antithrombotic therapy, results from a consecutive retrospective cohort

**DOI:** 10.1186/s13049-025-01431-1

**Published:** 2025-09-10

**Authors:** Aipi Forsberg-Puckett, Gabriele Wurm Johansson, Sara Regnér

**Affiliations:** 1 Department of Clinical Sciences, Malmö, Section of Surgery, Lund University, Malmö, Sweden; 2 Department of Surgery, Malmö, Skåne University Hospital, Malmö, Sweden; 3 Department of Surgery, Ystad Hospital, Ystad, Sweden; 4 Department of Clinical Sciences, Malmö, Section of Gastroenterology, Skåne University Hospital, Lund University, Malmö, Sweden

**Keywords:** Gastrointestinal bleeding, Antithrombotic therapy, Bleeding symptoms

## Abstract

**Background:**

Antithrombotic treatment might affect bleeding symptoms, identification of bleeding source and treatment for patients with acute gastrointestinal bleeding. This study aims to investigate possible differences in initial bleeding symptoms, identified bleeding site and treatment of patients with or without antithrombotic medication admitted for gastrointestinal bleeding.

**Methods:**

All consecutive adult patients primarily admitted for gastrointestinal bleeding at Skane University Hospital between 2018-01-01 and 2019-06-31, were included in this study. Data was retrospectively extracted from medical files. Patients were stratified according to antithrombotic therapy (antiplatelet or oral anticoagulants) on admittance or not. Groups were compared using Fisher’s exact test, Mann Whitney U-test, Kruskal-Wallis test and logistic regression analysis, including interaction models.

**Results:**

585 patients were included. Median age was 75 years and a majority (58%) were male. In total, 269 (46%) patients had no antithrombotic medication and 316 (54%) had some kind of antithrombotic medication. Patients with antithrombotic therapy had a higher age and Charlson comorbidity index than those without antithrombotic therapy. However, comparing patients with anticoagulants, antiplatelet medication and no antithrombotics, hemoglobin at arrival (median (interquartile range (IQR)) 99(75–130), 103(85–125) and 100(80–128) respectively, *p* = 0.851) and Shock index (0.65 (0.50–0.83), 0.67(0.53–0.81) and 0.66 (0.57–0.80) respectively, *p* = 0.529) did not differ between the groups. Patients with antithrombotic therapy more often presented with hematochezia alone (35.3% and 32.2% for anticoagulant and antiplatelet medications, respectively, 23% for those with no antithrombotics *p* = 0.017) and less frequently with hematemesis compared to patients with no antithrombotic therapy (14.7% and 24.6%, respectively, 33.1% in those with no antithrombotics, *p* < 0.001). Predicted probabilities of receiving endoscopic treatment, need for transfusion, and number of units transfused did not differ between groups.

**Conclusions:**

Patients with anticoagulant therapy more often present with a lower source of Gastrointestinal (GI) bleeding than both those on antiplatelet medications and those with no antithrombotics. However, the presentation of bleeding is similar regardless of any antithrombotic medication or not.

**Trial registration:**

ClinicalTrials.gov (NCT05195697), 19/01/2022.

**Supplementary Information:**

The online version contains supplementary material available at 10.1186/s13049-025-01431-1.

## Background

Gastrointestinal (GI) bleeding is a common reason for visits to emergency units. Although GI bleeding can be divided into upper and lower, the location of bleeding is not always obvious when the patient presents at the emergency department. The specific bleeding symptoms provide valuable information that leads to a suspicion of bleeding site and guides initiation of treatment; hematemesis (red or coffee-grounds like vomit) being considered a strong indicator of an upper bleeding source and hematochezia (bloody stool) often indicating a lower bleeding source. However, melena can be indicative of either an upper or lower GI bleeding source [1]. The mortality rate of GI bleeding varies between 3.9 and 14% [2, 3, 4, 5]. Independent risk factors for higher mortality in GI bleeding include severe comorbidities, high age, and male sex [2, 3, 4, 6].

Use of antithrombotic drugs affect the incidence as well as the treatment of GI bleeding. For many years, warfarin was the predominant anticoagulant used for prevention of thromboembolic events, but with the introduction of direct oral anticoagulants (DOACs) there has been a change in anticoagulant therapy. DOACs show a significant risk reduction for intracranial hemorrhage compared to warfarin, however, data on the occurrence of GI hemorrhage during DOAC therapy varies [7, 8]. Holster et al. describe DOACs as associated with a modest, but significantly higher, risk of GI bleeding compared with current standard care both in treatment for venous thromboembolism and prophylaxis for atrial fibrillation and acute coronary syndrome [8, 9].

Antiplatelet therapy is an important part in the treatment and prevention of coronary artery disease, peripheral vascular disease, and cerebrovascular disease. Treatment with antiplatelet medication has been shown to increase the risk of GI bleeding [10]. Antiplatelets can be taken both as a monotherapy or in combination with other antiplatelet drugs or DOACs [11].

The site of bleeding has in a previous study been shown to differ between types of antithrombotic therapy, with patients on DOACs more often presenting with a lower GI bleeding source than other antithrombotics [12]. The severity of GI bleeding in patients with antithrombotic therapy including DOACs is not well studied. One study suggested bleeding to be less severe in patients taking DOACs, also including patients not admitted to hospital [13]. The need for more data on how antithrombotic therapy affects patients with gastrointestinal bleeding is reflected in how it is implemented in risk stratification scores recommended in many guidelines [14, 15]. Though several of these scoring systems include comorbidities, few take into account the antithrombotic medications of the patient, and to a varying degree; the NOBLADS score include non-aspirin antiplatelet medications, whereas Strate only include aspirin use [16]. However, the impact of modern anticoagulant and antiplatelet treatment on initial symptoms, treatment and outcome of GI bleeding is not thoroughly studied. This study aims to investigate possible differences in patients treated with or without oral antithrombotic drugs on presenting symptoms, identification of bleeding source and treatment, using an unselected consecutive cohort of patients admitted to hospital with GI bleeding. More knowledge on this subject might aid clinicians in the emergency setting when these patients present with gastrointestinal bleeding of unknown origin.

## Materials and methods

All adult patients (18 years or older) admitted with GI bleeding at Skane University Hospital between 2018-01-01 and 2019-06-31, were retrospectively included in the study. Patients admitted through the emergency unit were identified using the primary diagnosis on discharge, including several ICD-codes that potentially could present with GI bleeding (supplementary Table 1). From this larger cohort, all patients primarily admitted for suspected GI bleeding were validated through a thorough review of the medical files and included in the study. Patients were stratified into groups based on antithrombotic therapy on admittance. Patients were treated according to local hospital routines.

### Data collection and statistical analysis

The study was performed in accordance with RECORD and STROBE guidelines for observational cohort studies [17]. Data on patient characteristics, treatment and outcome during the hospital stay was retrieved from medical records using a pre-defined protocol. Charlson comorbidity index (CCI) was used to consolidate the data for comorbidity [18, 19]. Shock index was calculated using the pulse and systolic blood pressure on arrival. Shock was defined as shock index > 1 or, in one patient, cardiac arrest [20].

The bleeding presentation at admission either reported by the patient or observed by healthcare or emergency personnel was recorded. Melena was defined as black or tarry stools, hematochezia as red blood in the stool and hematemesis as red blood or coffee-grounds-like vomit. Patients could have more than one visible sign of bleeding at admission. For statistical analysis visible bleeding was grouped into 3 bleeding categories as follows: Hematemesis group (hematemesis +- melena and/or hematochezia), Melena group (melena -+ hematochezia) or Hematochezia group (hematochezia only).

For statistical analysis SPSS version 28 (IBM Corporation, Armonk, NY, USA) or R (version 4.3.3) was used. Comparison of categorical variables was done using Fisher’s exact test. For continuous values group comparisons were made using the Mann-Whitney U or the Kruskal-Wallis nonparametric test. All group comparisons were unpaired. A *p*-value of ≤ 0.05 was considered statistically significant.

Logistic regression analysis was performed comparing patients with no antithrombotic medication with those with the different types of oral antithrombotic medication. Odds ratio (OR) was presented with a 95% confidence interval and *p*-value. Directed acyclic graphs were used to identify possible confounding factors [21] used for calculation of adjusted OR (OR^a^), where each model was adjusted for sex and CCI. Logistic regression was also used to explore interaction effects between visible bleeding source and identification of bleeding source and treatment. The results were presented as OR with 95% confidence intervals. Predicted probabilities were calculated and graphically presented with 95% confidence interval.

### Ethical considerations

The study was approved by the Regional Ethics Committee at Lund University (Dnr 2019–03583) and registered in ClinicalTrials.gov (NCT05195697).

## Results

### Clinical characteristics

Patient baseline characteristics can be seen in Table 1. A total of 585 patients were enrolled. In total, 269 (46%) patients had no antithrombotic medication and 316 (54%) had some kind of antithrombotic medication. Of those, 136 were anticoagulants (DOACs or warfarin), 171 were antiplatelet medications and 9 had a combination of anticoagulants and antiplatelet drugs. Since the group with combination therapy anticoagulant and antiplatelet medication was very small (*n* = 9) it was not used for statistical analysis.

**Table 1:** Baseline data

		All patients	No-Antithrombotic therapy	Single antithrombotic therapy		Combination antithrombotic therapy	*p*-value^†^
				Anticoagulant	Antiplatelet		
*n*		585	269 (46%)	136 (23.2%)	171 (29.2%)	9 (1.5%)	
Age, median (IQR)		75 (64–83)	68 (53–78)	82 (74–86)	76 (70–83)	77 (72–86)	< 0.001
Sex, *n* male (% male)		338 (57.8%)	159 (59.1%)	74 (54.4%)	98 (57.3%)	7 (77.8%)	0.665
BMI, median (IQR)		25.9 (23.0-29.3)	25.5 (22,7-28.7)	26.2 (22.9–31.1)	26.4 (23.4–29.5)	28.3 (22.8–31.7)	0.073
CCI, median (IQR)		4 (2–6)	3 (1–4)	5 (3–7)	5 (3–6)	7 (4.5-7)	< 0.001
Hb*, median (IQR)		101 (81–127)	100 (80–128)	99 (75–130)	103 (85–125)	82 (70–102)	0.851
Shock index, median (IQR)		0.65 (0.54–0.81)	0.66 (0.57–0.80)	0.65 (0.50–0.83)	0.67 (0.53–0.81)	0.65 (0.52–0.69)	0.529
Shock on arrival, *n* (%)		64 (10.9%)	29 (10.8%)	18 (13.2%)	16 (9.4%)	2 (22.2%)	0.535
Arrival bleeding symptoms**	Hematemesis	152 (26%)	89 (33.1%)	20 (14.7%)	42 (24.6%)	1 (11.1%)	< 0.001
	Melena	320 (54.7%)	150 (55.8%)	72 (52.9%)	92 (53.8%)	6 (66.7%)	0.844
	Hematochezia	260 (44.4%)	102 (37.9%)	72 (52.9%)	82 (48%)	4 (44.4%)	0.009
Visible bleeding categories***	Hematemesis group	152 (26%)	89 (33.1%)	20 (14.7%)	42 (24.6%)	1 (11.1%)	< 0.001
	Melena group	239 (40.9%)	105 (39%)	60 (44.1%)	68 (39.8%)	6 (66.7%)	0.601
	Hematochezia group	167 (28.5%)	62 (23%)	48 (35.3%)	55 (32.2%)	2 (22.2%)	0.017
	Uncertain	27 (4.6%)	13 (4.8%)	8 (5.9%)	6 (3.5%)	0	0.613

BMI = Body Mass Index, CCI = Charlson Comorbidity Index, IQR = Interquartile range, *Hb = hemoglobin on arrival(g/L), ** Visible bleeding on arrival (multiple possible per patient) *** Bleeding categories: Patients stratified into one of Hematemesis group (hematemesis +- melena and/or hematochezia), Melena group (melena -+ hemoatochezia) or Hematochezia group (hematochezia only). ^†^All analysis was done excluding combination therapy group. Analysis was done using Kruskal-Wallis or Chi^2^-test

The distribution of hemoglobin value on arrival did not differ significantly between the groups (*p* = 0.851). Initial circulatory status evaluated as shock index and proportion of patients in shock on arrival (shock index > 1 or cardiac arrest) was also similar in different groups. Distribution of age differed between the groups (*p* < 0.001). The mean age of patients treated with antithrombotic therapy was higher compared to patients with no treatment; highest in patients with anticoagulants (80 years). The distribution of CCI also differed between the groups (*p* < 0.001); patients with no antithrombotic medications had fewer comorbidities, with a median value of 3 compared to 5 in the groups with antithrombotics.

### Presentation of bleeding

Presentation of bleeding as reported by the patient or observed by healthcare or emergency personnel is described in Table 1.

When grouped into categories, the distribution of bleeding symptoms varied between different antithrombotic therapies (Table 1) (*p* = 0.008). In patients with no antithrombotic therapy, 33.1% presented with hematemesis (with or without either hematochezia or melena), compared to 14.7% in those with anticoagulants and 24.6% in those with antiplatelet therapy (*p* < 0.001). The proportion of patients with melena (with or without hematochezia) was similar in the different treatment groups. Hematochezia alone was slightly less common in patients with no antithrombotic therapy (23%) compared to 35.3% and 32.2% for anticoagulant and antiplatelet medications, respectively (*p* = 0.017). When analyzed with logistic regression adjusted for CCI and sex, the odds of presenting with hematemesis (with or without melena) were lower in patients with anticoagulant therapy compared to both those with no antithrombotic medication and those with antiplatelets (*p* < 0.001 and *p* = 0.035 respectively) (Table 2). In 27 patients bleeding was recorded in the medical file as “visible” but not further described. The proportion of these patients in the different treatment groups did not differ significantly and they were excluded from any statistical analysis comparing bleeding symptoms.

**Table 2:** The odds ratio of presenting with different bleeding symptoms for patients with different antithrombotic medications, adjusted for CCI and sex. (*= *p* < 0.05)

	Anticoagulant vs. None OR (95% CI)	Antiplatelet vs. None OR (95% CI)	Antiplatelet vs. Anticoagulant OR (95% CI)
Hematochezia group	1.80 (1.12; 2.90)*	1.57 (0.99; 2.49)	0.92 (0.57; 1.50)
Melena group	1.36 (0.87; 2.13)	1.03 (0.67; 1.58)	0.78 (0.49; 1.24)
Hematemesis group	0.371 (0.21; 0.65)	0.75 (0.47; 1.19)	1.90 (1.05; 3.46)*

### Identification of bleeding source

Successful identification of the bleeding source in the total cohort including all bleeding symptoms, did not vary significantly between treatment. To analyze if antithrombotic drugs could affect both bleeding category and identification of bleeding source an interaction analysis was performed. This analysis indicated interaction effects, where the greatest difference was seen in the group with melena and/or hematochezia groups (supplementary Table 3, supplementary Fig. 1).

### Bleeding symptom relating to need for treatment

As expected, the distribution of patients needing endoscopic intervention differed significantly between the visible signs of bleeding categories, with a higher need for intervention in the patients with hematemesis (30.9%), followed by melena (23%) and hematochezia (14%) (*p* = 0.006). Need for surgical or interventional radiology intervention followed the same trend; 4.6% of those with hematemesis, 2.9% of those with melena and 1.8% with hematochezia, but did not show statistically significant difference (*p* = 0.342). The same trend was also seen when comparing all-cause in-hospital mortality where patients with hematemesis had a mortality rate of 5.9%, those with melena 2.9% and hematochezia 1.8% (*p* = 0.111).

**Fig. 1:**
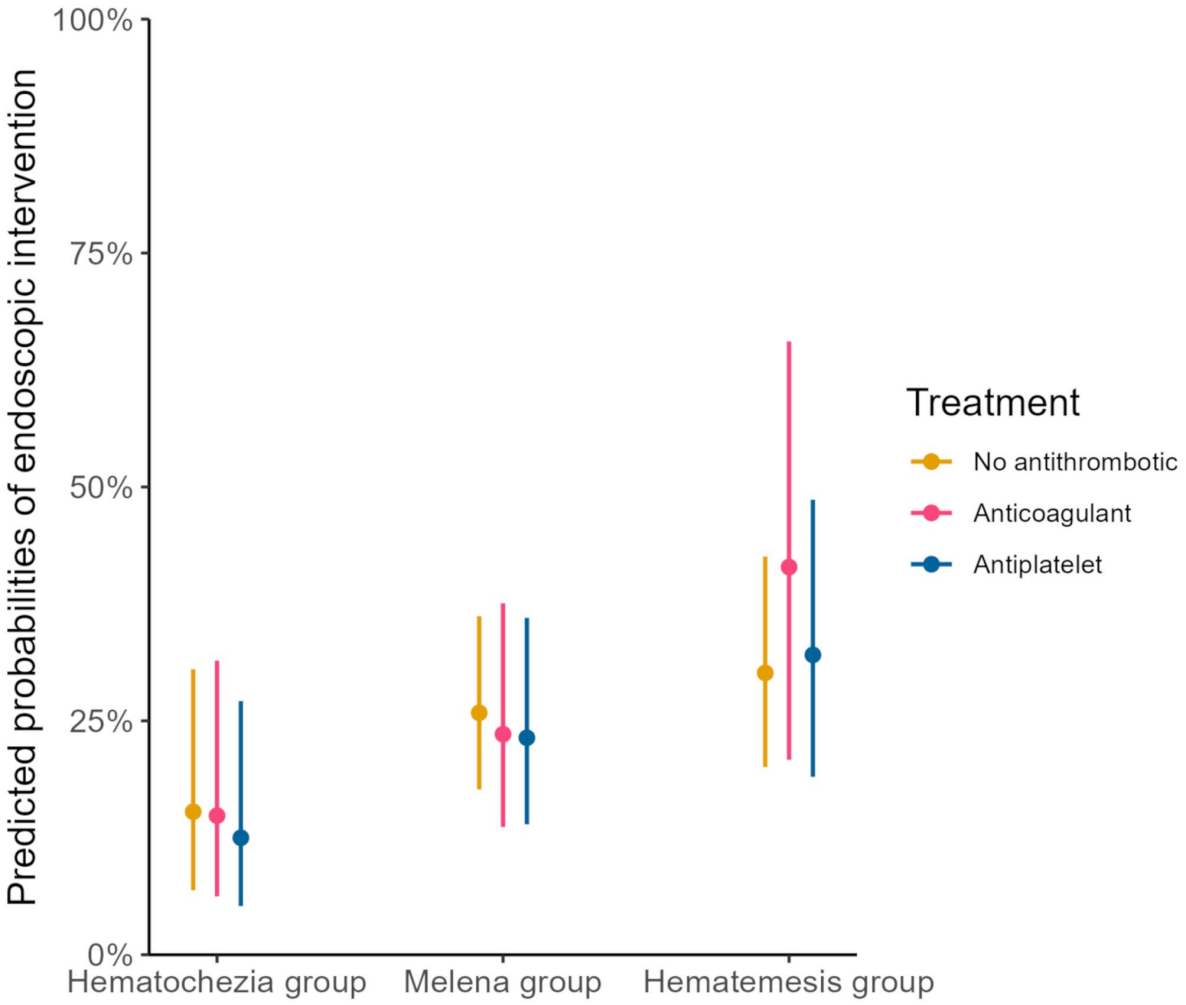
Predicted probabilities of receiving endoscopic treatment, adjusted for sex and CCI (lines indicating 95% confidence interval)

**Fig. 2:**
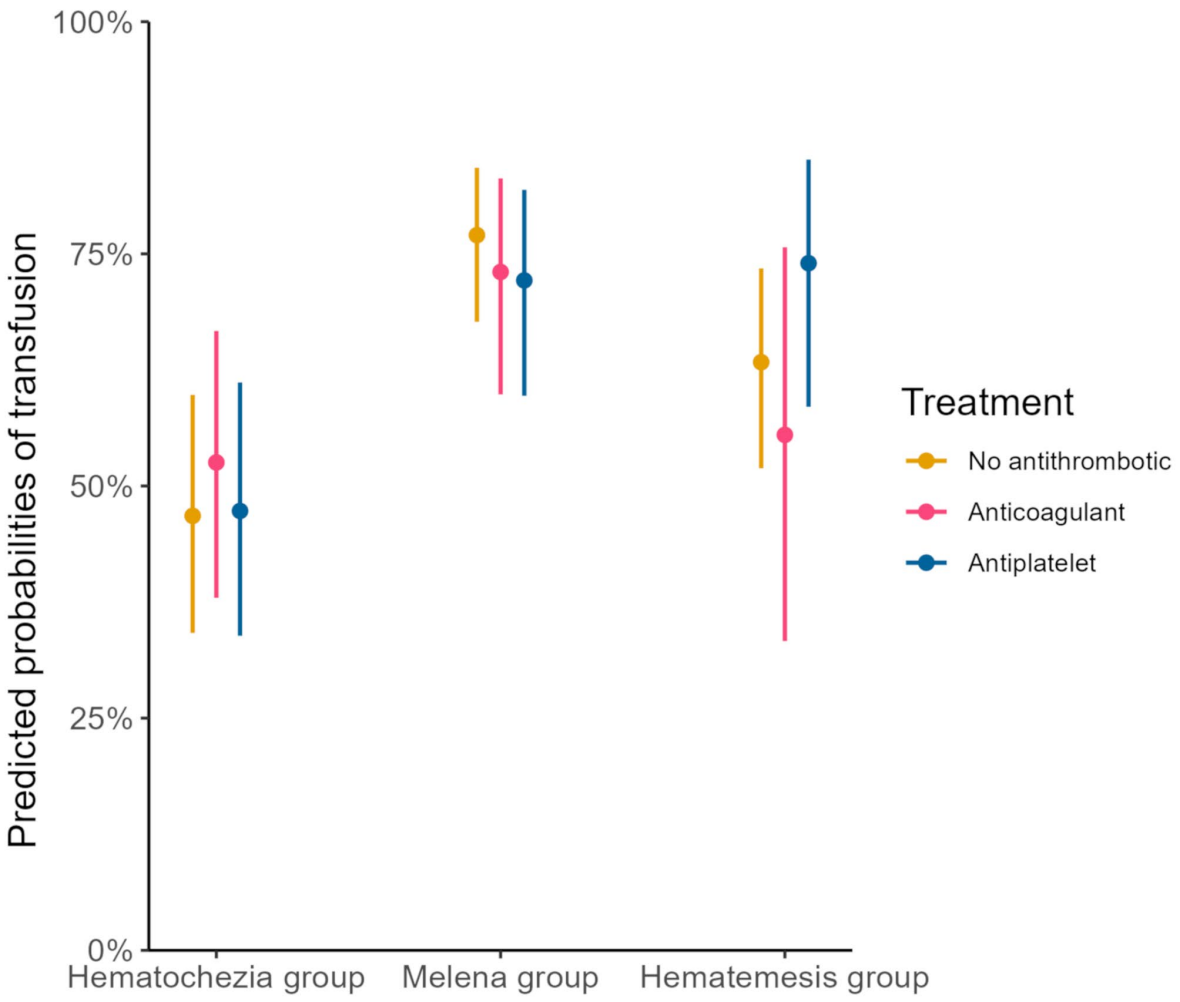
Predicted probabilities of transfusion, adjusted for sex and CCI (lines indicating 95% confidence interval)

When comparing patients with antithrombotic- anticoagulant- and no antithrombotic therapy in the total cohort, no difference was found in either need for erythrocyte transfusion, endoscopic treatment, surgery/IR or mortality within the different bleeding categories (**data not shown**). When stratified in bleeding categories, the predicted probabilities of receiving endoscopic treatment, varied somewhat between the different bleeding categories, but was similar in all three treatment groups (Fig. 1). This was true also in the need for transfusion, and number of units transfused (Fig. 2).

## Discussion

In this study we attempted to describe and analyze possible differences in the presentation of GI bleeding, identification of bleeding source and outcome of patients with or without antithrombotic medication presenting at the emergency unit. Though initial bleeding symptoms varied in frequency in relation to different medication groups, it did not correlate with the need for different treatments or mortality of the patients in this cohort.

Use of antithrombotic therapies have changed over the last 10–15 years. From the emergency doctor’s perspective there is a need to understand if the patients seeking emergency care for GI bleeding have similar characteristics as earlier or if the population has changed related to different medications. With an aging population, a larger part of the population may be prescribed antithrombotic drugs in the future. In the unselected consecutive cohort in the present study, a majority of patients (54%) had antithrombotic or anticoagulant medication on admittance, compared to around 12% in the general population at the time [22]. The figures are similar in earlier published data; for instance, around 42% of the population used by Laursen et al. developing the ABC-score had some form of antithrombotic medication [23]. This indicates the relevance of studies in this field. Differences between patients with and without antithrombotic medications could affect initial risk evaluation or diagnostic work initiated for the patient at the emergency unit. Although not thoroughly studied in this setting, other studies have indicated differences between patients with antithrombotic treatments [4].

In the current study, patients with antithrombotic drugs did not differ from other patients in initial bleeding related parameters including hemoglobin value and shock index. This interesting finding indicates that similar initial risk assessment and treatment can be applied to these patients in general. However, this does not replace the patient-specific risk assessment done by the physician at the emergency unit. In a study of upper GI bleeding, Thiebaud et al. noted that use of antithrombotic medication was not a predictor for mortality [5]. Still, the difference in bleeding distribution, where patients without antithrombotic drugs had hematemesis to a higher extent and patient with antithrombotic medications more often show hematochezia, indicate that antithrombotic drugs affect the bleeding site and that the cohort of patients with antithrombotic drugs differ from patients without these medications. The following analysis in this study aimed to understand if this difference has clinical implications.

One interesting finding is that the proportion of patients with an identified bleeding source was similar for patients with and without antithrombotic medication when the total cohort was analyzed. However, when comparing different antithrombotics the primary bleeding source was found in a smaller percentage of patients in the anticoagulant group than both the antiplatelet group and those with no antithrombotics. Previous studies have suggested an effect of DOACs is “unmasking” colorectal tumors by allowing for earlier detection either by inducing visible bleeding or with a positive fecal hemoglobin test [24]. It has previously been hypothesized that active drug left in the intestinal lumen in patients with DOACs may exacerbate any bleeding occurring in the bowel, particularly colon [25]. Supporting the hypothesis that anticoagulant therapy might induce small, more often self-limiting but initially visible bleeds are the findings of this study, as the bleeding source was less often identified in patients with anticoagulant therapy without affecting outcome. However, this study does not take into account any pro-coagulant agents used in the care of these patients and the retrospective design inherits risk for bias. Thus results should be interpreted accordingly.

When stratifying patients related to identified bleeding source, results differ between upper and lower GI bleeding. There was a difference in how a upper GI bleeding presented, with those with no antithrombotics having a higher rate of hematemesis than those on either antiplatelet or anticoagulant therapy, who both more commonly presented with melena. However, a lower source of GI bleeding presented very similarly with different antithrombotic therapies. In patients who were later found to have a lower GI bleeding source, the majority had hematochezia on presentation regardless of antithrombotic therapy or not.

The retrospective design of this study inherits some disadvantages. Even if there were no study protocols for diagnostic and therapeutic interventions, all patients were treated according to similar hospital routines. However, we cannot exclude that patients might be treated differently, introducing bias. Collecting the data from medical records also relies on the accuracy of the information recorded by the treating physician, and some data may be missing. There may also be some cases of GI bleeding that have been miscoded in the medical files, and therefore not included in the study. However, to mitigate this the initial search was broad (supplementary Table 1). In this study the data set was complete except for recorded BMI, which was not always available. One advantage of the retrospective design is describing an unselected consecutive cohort as when patients present in the emergency department. The total results are in line with earlier studies indicating the results are representative for these patients [26, 27]. However as it is a retrospective study, all results must be interpreted with caution.

## Conclusions

Patients with anticoagulant therapy more often present with a lower source of GI bleeding than both those on antiplatelet medications and those with no antithrombotics. This should be taken into account when initially evaluating these patients. Overall patients on anticoagulants are also less likely to present with hematemesis, even with a later confirmed upper GI bleeding. Furthermore, results indicate that the need for endoscopic interventions and transfusions are dependent on initial presenting symptoms but not affected by antithrombotic therapy at admission.

## Electronic supplementary material

Below is the link to the electronic supplementary material.

Supplementary Material 1

## Data Availability

The datasets generated and/or analyzed during the current study are not publicly available due to the confidential nature of the material but are available from the corresponding author upon reasonable request.

## Abbreviations

BMI: Body Mass Index
CCI: Charlson Comorbidity Index
DOAC: Direct oral anticoagulant
GI: Gastrointestinal
UGIB: Upper gastrointestinal bleed
LGIB: Lower gastrointestinal bleed
IR: Interventional radiology
OR: Odds ratio
OR^a^: Adjusted odds ratio
