# Supplementary material for: Presentation of gastrointestinal bleeding in patients with antithrombotic therapy, results from a consecutive retrospective cohort
Source: Scand J Trauma Resusc Emerg Med. 2025 Sep 10;33:146. doi: 10.1186/s13049-025-01431-1 (PMC12421751; doi:10.1186/s13049-025-01431-1)
Supplement: Supplementary file 1 — Supplementary Material 1 [file 13049_2025_1431_MOESM1_ESM.docx]

**Supplementary Table 1:** Clinical Conditions and Corresponding ICD-10 Codes Used for Patient Selection

| **Clinical condition** | **ICD-10** |
| --- | --- |
| Gastro-oesophageal laceration-hemorrhage syndrome | K226 |
| **Gastric ulcer with hemorrhage** | K250, K252, K254, K256 |
| **Duodenal ulcer with hemorrhage** | K260, K262, K264, K266 |
| Peptic ulcer, site unspecified, with hemorrhage | K270, K272, K274, K276 |
| Hemorrhage of anus and rectum | K625 |
| Gastrointestinal hemorrhage, unspecified | K922 |
| **Oesophageal varices, with hemorrhage** | I982-I983 |
| Malignant neoplasm of oesophagus | C15X |
| Malignant neoplasm of stomach | C16X |
| Malignant neoplasm of small intestine | C17X |
| Malignant neoplasm of colon | C18X |
| Malignant neoplasm of rectosigmoid junction | C19X |
| Malignant neoplasm of rectum | C20X |
| Malignant neoplasm of anus and anal canal | C21X |
| Malignant neoplasm of intestinal tract, part unspecified | C260 |
| Malignant neoplasm of ill-defined sites within the digestive system | C269 |

**Supplementary Table 2:** Presenting bleeding symptoms

| **Identified bleeding source** | **Visible bleeding symptoms** |  | **All patients** | **No antithrombotic therapy** | **Single antithrombotic therapy** | | **Combination antithrombotic therapy** |
| --- | --- | --- | --- | --- | --- | --- | --- |
|  |  |  |  |  | **Anticoagulant** | **Antiplatelet** |  |
|  | **All bleeding symptoms** | | 286(48.9%) | 150(55.8%) | 47(34.6%) | 89(52%) | 0 |
| **UGIB** |  | **Hematemesis** | 109(38.1%) | 63(42%) | 13(27.7%) | 33(37.1%) | 0 |
|  |  | **Melena** | 137(47.9%) | 65(43.3%) | 26(55.3%) | 46(51.7%) | 0 |
|  |  | **Hematochezia** | 20(7%) | 11(7.3%) | 4(8.5%) | 5(5.6%) | 0 |
|  |  | **Uncertain** | 20(7%) | 11(7.3%) | 4(8.5%) | 5(5.6%) | 0 |
|  |  |  |  |  |  |  |  |
|  | **All bleeding symptoms** | | 117(20%) | 45(16.7%) | 39(28.7%) | 32(18.7%) | 1(11.1%) |
| **LGIB** |  | **Hematemesis** | 2(1.7%) | 1(2.2%) | 1(2.6%) | 0 | 0 |
|  |  | **Melena** | 31(26.5%) | 13(28.9%) | 9(23.1%) | 9(28.1%) | 0 |
|  |  | **Hematochezia** | 82(70.1%) | 31(68.9%) | 27(69.2%) | 23(71.9%) | 1(100%) |
|  |  | **Uncertain** | 2(1.7%) | 0 | 2(5.1%) | 0 | 0 |
|  |  |  |  |  |  |  |  |
|  | **All bleeding symptoms** | | 182(31.1%) | 74(27.5%) | 50(36.8%) | 50(29.2%) | 8(88.9%) |
| **Unidentified^*^** |  | **Hematemesis** | 41(22.5%) | 25(33.8%) | 6(12%) | 9(18%) | 1(12.5%) |
|  |  | **Melena** | 71(39%) | 27(36.5%) | 25(50%) | 13(26%) | 6(75%) |
|  |  | **Hematochezia** | 65(35.7%) | 20(27%) | 17(34%) | 27(54%) | 1(12.5%) |
|  |  | **Uncertain** | 5(2.7%) | 2(2.7%) | 2(4%) | 1(2%) | 0 |

*Bleeding source not identified during hospital stay.

^†^All analysis was done with Chi^2^ test and excluding combination therapy group.

**Supplementary Table 3**: Interaction analysis for identification of bleeding source in groups with different antithrombotic treatments and different bleeding categories, adjusted for CCI and sex.

|  | **Anticoagulant vs None OR (95% CI)** | **Antiplatelet vs None OR (95% CI)** | **Antiplatelet vs Anticoagulant OR (95% CI)** |
| --- | --- | --- | --- |
| **Hematochezia group** | 0.91 (0.41; 2.04) | 0.53 (0.25; 1.13) | 0.58 (0.26; 1.28) |
| **Melena group** | 0.55 (0.27; 1.10) | 1.66 (0.78; 3.57) | 3.04 (1.37; 6.74) |
| **Hematemesis group** | 1.05 (0.36; 3.08) | 1.50 (0.62; 3.64) | 1.43 (0.42; 4.84) |

**Supplementary figure 1:** Predicted probabilities of bleeding source being identified, adjusted for sex and CCI


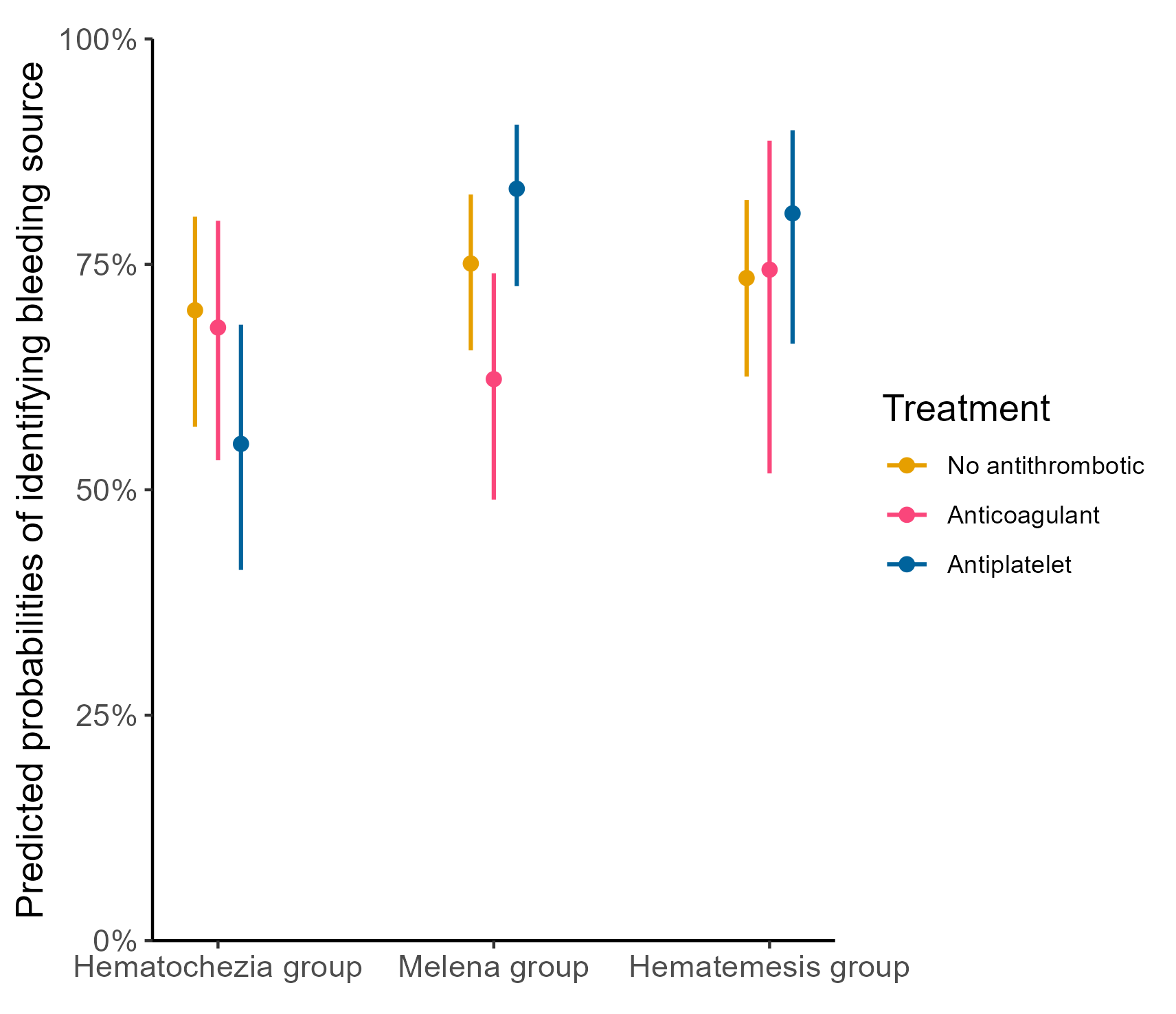


*Presentation relating to antithrombotic therapy by identified bleeding source*

Upper gastrointestinal bleed (UGIB) and Lower gastrointestinal bleed (LGIB)

Since many studies investigate cohorts where bleeding source is already identified as either UGIB or LGIB we decided to analyze antithrombotic treatments in relation to identified bleeding source. An identified UGIB was less common in patients with anticoagulants (34.6%) compared to 52% with antiplatelets (p=0.012) and 55.8% with no antithrombotics (p=0.001). A LGIB source was more common in patients with anticoagulant therapy (28.7%) than in both patients with antiplatelet medication (18.7% p=0.065), and those without (16.7% p=0.005) (**Supplementary figure 2, supplementary table 2**).

In the interaction analysis investigating both initial bleeding symptoms and antithrombotic medications in patients with identified UGIB or LGIB respectively, there was no significant difference in the distribution of symptoms in relation to antithrombotic medications.

**Supplementary figure 2:** Presentation of bleeding by identified bleeding source


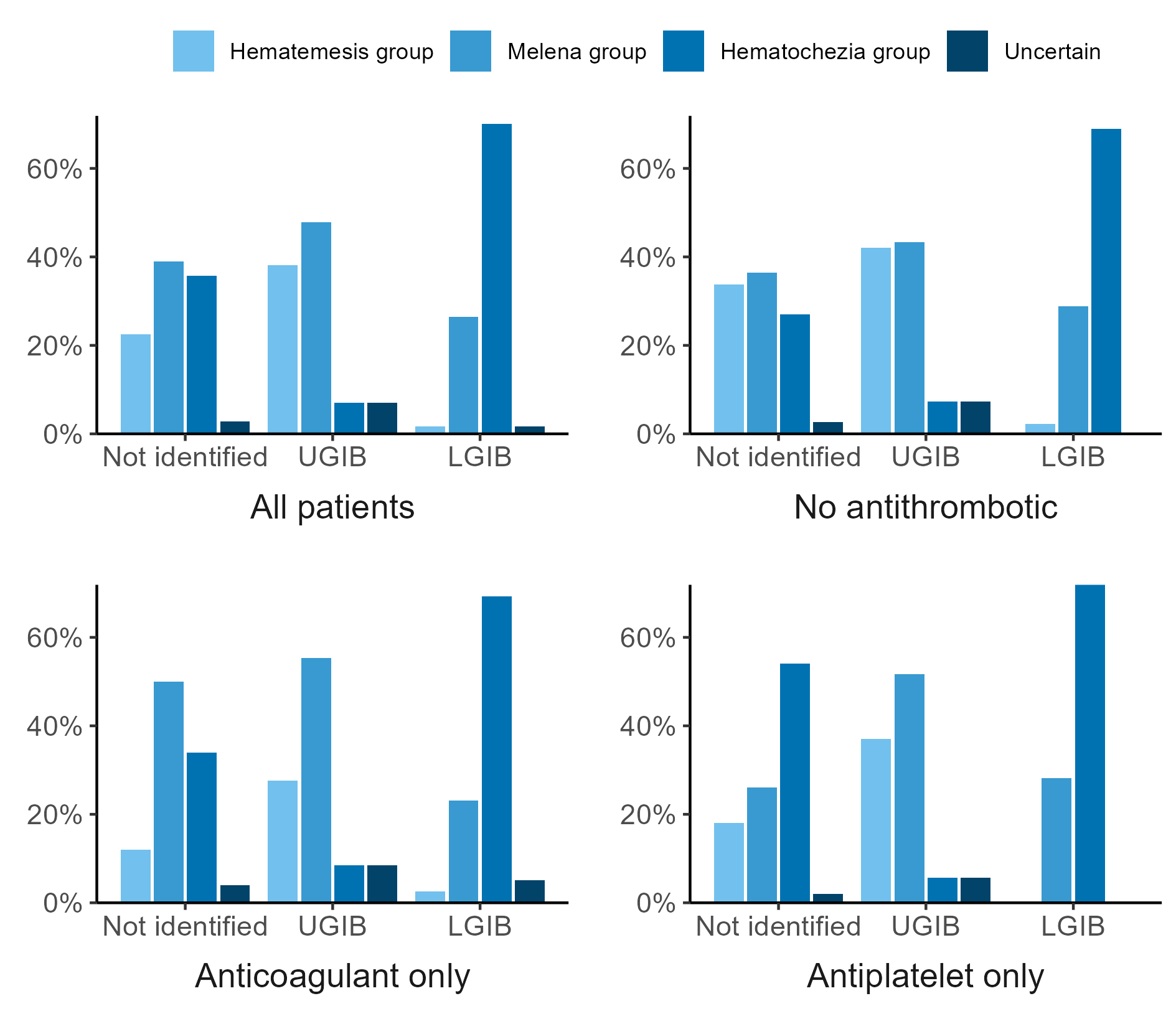


No identified bleeding source

When comparing patients in whom the bleeding source was not identified during the hospital stay, there was a statistically significant difference in the distribution of symptoms between the treatment groups. Hematemesis (with or without melena or hematochezia) was present in 33.8% of those with no antithrombotic treatment, but only 12% and 18% in those with anticoagulants and antiplatelets, respectively (p=0.022). Melena (with or without hematochezia) was, instead, more common in those with anticoagulants (50%) compared to 36.5% in those with no treatment and 26% in those with antiplatelets (p=0.13). Hematochezia alone was found more commonly in patients with antiplatelet treatment (54%) compared to 27% in those with no treatment and 34% in those with anticoagulants (p=0.009).
